# Analysis of FFR Measurement Clinical Impact and Cost-Effectiveness Compared to Angiography In Multi-Arterial Patients Undergoing PCI

**DOI:** 10.5935/abc.20180261

**Published:** 2019-01

**Authors:** Fernando Mendes Sant'Anna, Lucas Bonacossa Sant'Anna

**Affiliations:** 1 Universidade Federal do Rio de Janeiro, Campus Macaé, Macaé, RJ - Brazil; 2 Serviço de Hemodinâmica da Clínica Santa Helena, Cabo Frio, RJ - Brazil; 3 Fundação Técnico-Educacional Souza Marques, Rio de Janeiro, RJ - Brazil

**Keywords:** fractional Flow Reserve,Myocardial, Cost-Benefit Analysis, Coronary Artery Disease/economics, Angioplasty, Balloon Coronary, Stents

The study by Quintella et al.^1^ published in this issue of the journal, brings us valuable information about the use of an important physiological evaluation tool in the hemodynamic laboratory. FFR-guided treatment (myocardial fractional flow reserve), used in the percutaneous coronary intervention (PCI) with bare-metal stent (BMS) implantation in multi-arterial patients treated in the Unified Health System (SUS) has been shown to be useful in decreasing the incidence of new revascularization of the target vessel (clinical restenosis), as well as being cost-effective when compared to the angiography-guided treatment.

The value of FFR to predict major adverse cardiovascular events (MCAEs) prior to PCIs has been established for many years. Its ability to detect ischemia and, with this, to guide the most appropriate treatment, has undergone the test of time, and passed. The 15-year follow-up of the DEFER^2^ study in single-vessel patients, and the 5-year studies, FAME 1,^3^ and FAME 2,^4^ in multiarterial patients, showed consistent and unquestionable results, with a better, or at least similar, clinical progression, in the FFR-guided groups, using less stents with fewer lesions and consequently lower costs, as well as evidenced the safety of leaving lesions whose FFR was not indicative of ischemia only on drug treatment.

The limited value of angiography to predict ischemia has long been known. Sant'Anna et al.^5^ showed a weak correlation between angiography, expressed as a percentage of stenosis diameter (SD), and FFR (rho = - 0.33), especially in intermediate lesions (between 40% and 70%). This disagreement between SD and physiology has already been documented in several other studies, such as that by Toth et al.^6^ and Park et al.,^7^ which also showed disagreement rates between FFR and angiography of 36% and 39% respectively. In a study published in 2007,^8^ in 250 patients (452 lesions) assessed by FFR before PCI, 32% of the lesions had their initially planned treatment strategy modified after FFR measurement, which is a major change because it would imply inadequate treatment in more than one third of the patients. More recently, Ciccarelli et al.^9^ in a FAME 2 substudy, analyzed the value of angiography compared to FFR to predict the natural history of coronary lesions, correlating MCAE index with the angiographic and physiological importance of these lesions in patients (n = 607) who were initially left only on drug treatment. In the subgroups in which FFR was discordant of angiography (FFR > 0.80 and SD ≥ 50% or FFR ≤ 0.80 and SD < 50%), clinical progression was worse in those in whom FFR was ≤ 0, 80, even if the lesion was not significant, and benign in those in whom FFR was > 0.80, regardless of SD.

In the study by Quintella et al.,^1^ MCAE that was reduced in the FFR group was due to the need for new revascularization of the target vessel, with no difference in mortality or infarction. Even with the limited number of patients involved in the study, this data is in agreement with what was presented in the FAME studies, in which, after 5 years of progression, only the need for new revascularization remains different in the groups. We call the attention to the low rate of clinical restenosis in the FFR group (5.8%) of the study by Quintella et al.,^1^ because he used only BMS, which may be due to the fact that much less lesions were treated compared to the angio group (1.14 vs. 2.22 stents per patient), and with better selection criteria.

Another interesting finding of the study is the cost-effectiveness (CE) relationship, measured by the incremental cost-effectiveness ratio (ICER), which represents the ratio between the costs of technologies under analysis, and their effectiveness. This ratio is usually adjusted for quality of life, and expressed as QALY (quality-adjusted life year). Costs below USD 20,000/QALY are accepted to be highly supportive of the technology tested. The ICER calculated for the study by Quintella et al.^1^ was of R$ 21,156, 55, totally within the CE criteria, mainly if we consider that only BMS were used, that is, if DES were used, ICER would be even lower. Fearon et al.^10^ have published an interesting study on FFR CE in the population of FAME 1,^10^ in which the author points out that the FFR-guided strategy has a lower cost compared to that guided by angiography in 90.74%, and is cost-effective in 99.96% of cases, being one of those rare situations where a new technology not only improves outcomes, but also saves resources. Siebert et al.^11^ found similar findings in the Australian population, where 1.776 USD would also be saved per patient over 1 year with the use of FFR during PCI.

Although we cannot extrapolate these results from other countries to ours, because the prices practiced and the reimbursement system are different, we can still assume that now, when SUS begins to allow the use of drug-eluting stents at a more competitive price, the strategy of use of FFR becomes even more attractive.
